# Supplementary material for: Letting the World See through Your Eyes: Using Photovoice to Explore the Role of Technology in Physical Activity for Adolescents Living with Type 1 Diabetes
Source: Int J Environ Res Public Health. 2022 May 23;19(10):6315. doi: 10.3390/ijerph19106315 (PMC9140903; doi:10.3390/ijerph19106315)
Supplement: Supplementary file 1 [file ijerph-19-06315-s001.zip › ijerph-1690504-supplementary.pdf]

Supplementary Materials

Letting the World See Through Your Eyes: Using Photovoice to Explore the Role of Technology in Physical Activity for Adolescents Living with Type 1 Diabetes

Diane Morrow [1,*], Alison Kirk [2], Fiona Muirhead [2] and Marilyn Lennon [1]

This data is submitted to provide evidence of the coding process using photovoice and thematic analysis with co-production techniques as described in the methods section of the paper.

Coding analysis processes - photovoice

1. **Initial coding by researcher –** margin notes to discuss with co-researchers, also serving to train co-researchers in “what do codes look like?”.

2. **Co-production – analysis with participants.**
   Emailing or Zoom call with participants, discussing codes with participants with the following:

   P Describe your photo
   H What is happening in the photo?
   O Why take a photo Of this?
   T What does this photo Tell us?
   O How can this photo provide Opportunity to improve an experience?

Example on slide 4 – of first order coding, labelling with participants over video conferencing.

3. **Second cycle of coding, with assistance from memoing - Research journal / logbook**
   Observation Notes – written whilst talking on Zoom, and whilst familiarisation with data
   Theoretical Notes – hunches, connections, interpretations, critiques – (all in reflexive journal).
   Listening carefully to audio/video with participants.

4. **Research team discussion –** Sharing 100% of data to discuss coding process and agreements – sharing data and Nvivo maps.

Informal note taking – during participatory co-researcher data analysis. In manuscript of paper, see lines 194-198

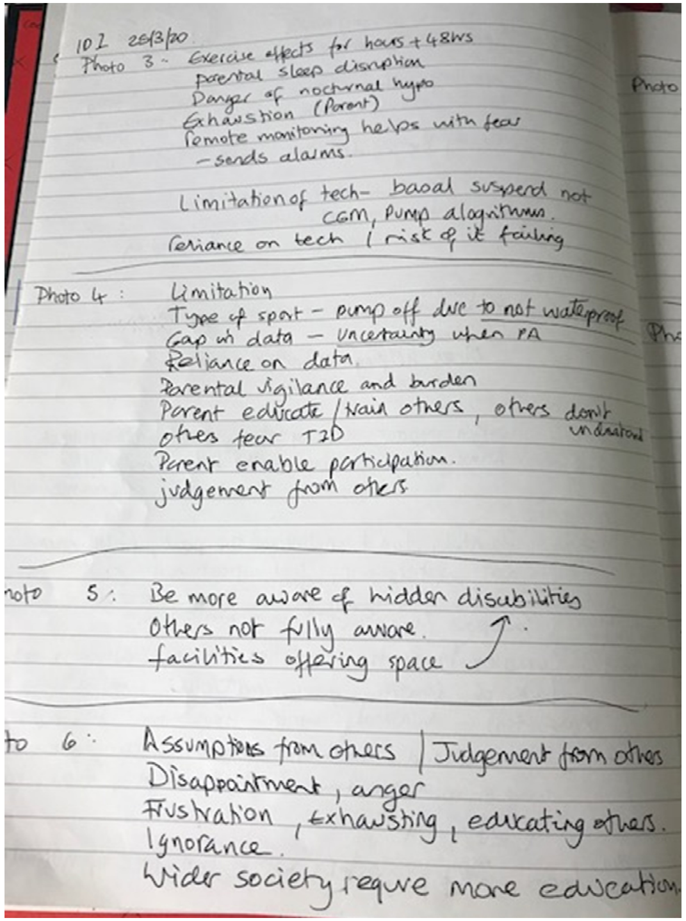

10L 26/3/20
Photo 3 - Exercise effects for hours + 48hrs
parental sleep disruption
Danger of nocturnal hypo
Exhaustion (Parent)
remote monitoring helps with fear
- sends alarms.

Limitation of tech - basal suspend not
CGM, Pump alogrithms.
reliance on tech / risk of it failing

Photo 4 : Limitation
Type of sport - pump off due to not waterproof
Gap in data - Uncertainty when PA
Reliance on data.
Parental vigilance and burden
Parent educate / train others, others don't understand
others fear T2D
Parent enable participation.
judgement from others

Photo 5 : Be more aware of hidden disabilities
Others not fully aware.
facilities offering space

Photo 6 : Assumptions from others | Judgement from others
Disappointment, anger
Frustration, exhausting, educating others.
Ignorance.
Wider society require more education.

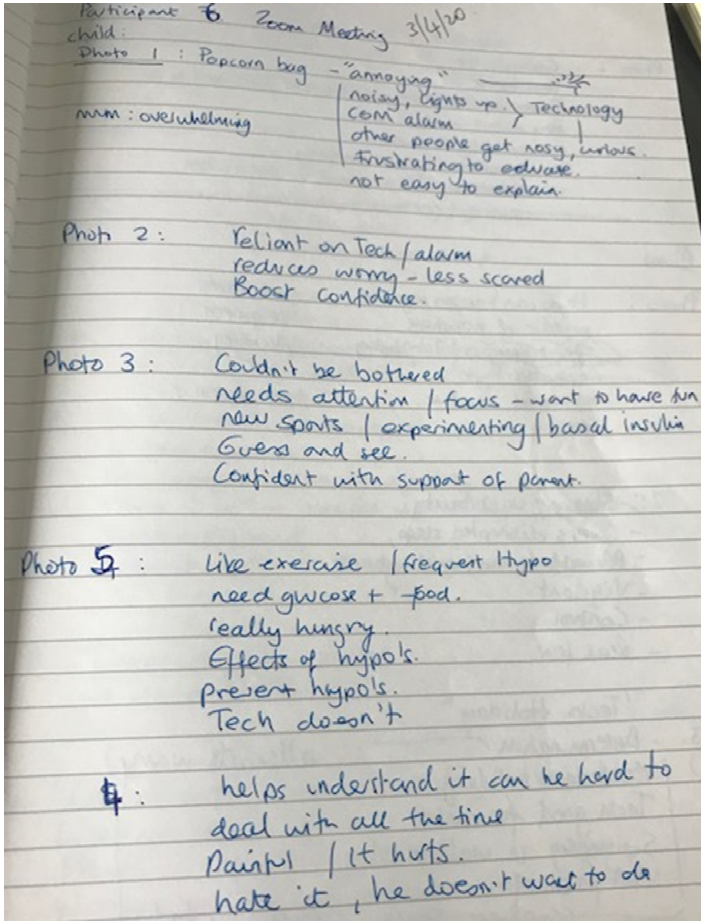

Participant 6 Zoom Meeting 3/4/20
child:
Photo 1 : Popcorn bag - "annoying"
noisy, lights up. } Technology
CGM alarm
other people get nosy, curious.
frustrating to educate.
not easy to explain.
mum : overwhelming

Photo 2 : reliant on Tech / alarm
reduces worry - less scared
Boost confidence.

Photo 3 : Couldn't be bothered
needs attention / focus - want to have fun
new sports / experimenting / basal insulin
Guess and see.
Confident with support of parent.

Photo 5 : Like exercise / frequent Hypo
need glucose + food.
really hungry.
Effects of hypo's.
prevent hypo's.
Tech doesn't

4 : helps understand it can be hard to
deal with all the time
Painful / it hurts.
hate it, he doesn't want to do

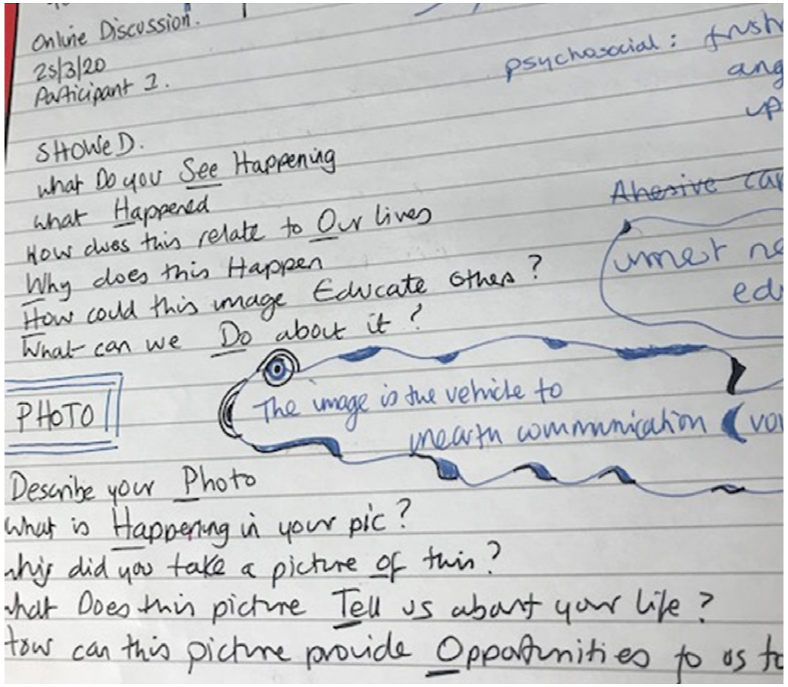

Online Discussion.
25/3/20
Participant 1.

SHOWeD.
what Do you See Happening
what Happened
How does this relate to Our lives
Why does this Happen
How could this image Educate others?
What can we Do about it?

PHOTO

The image is the vehicle to
mean[illegible] communication

Describe your Photo
What is Happening in your pic?
why did you take a picture of this?
what Does this picture Tell us about your life?
How can this picture provide Opportunities to us to [illegible]

psychosocial : frust[illegible]
ang[illegible]
up[illegible]

~~Ahesive ca[illegible]~~
unmet ne[illegible]
ed[illegible]

Coding process continued onto next slide: using Nvivo, labelling and coding using a combination of participatory codes and non-participatory codes. See lines 206-215 in manuscript for deeper explanation.

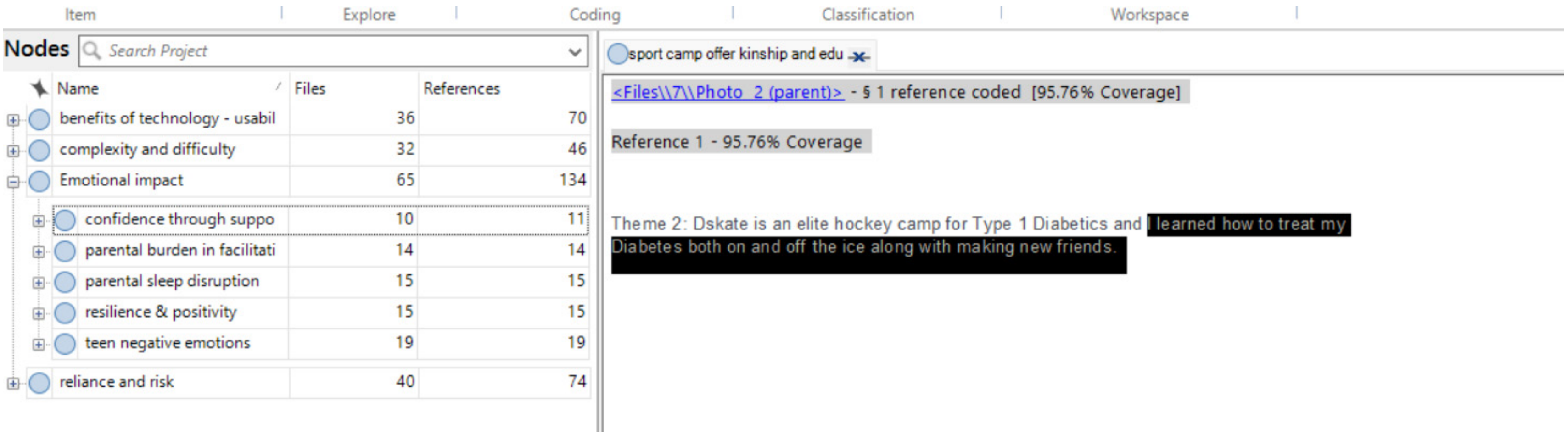

Nodes Search Project

Name	Files	References
benefits of technology - usability & psychosocial	36	70
complexity and difficulty	32	46
Emotional impact	65	134
confidence through support	10	11
parental burden in facilitation	14	14
parental sleep disruption	15	15
resilience & positivity	15	15
teen negative emotions	19	19
reliance and risk	40	75

Drag selection here to code to a new node

reliance and risk

References 1-2 - 41.50% Coverage

I didn't hear my pump alarm. Turns out I was extremely hypo.

<Files\\1\\Photo 5 voice (child)> - § 2 references coded [90.80% Coverage]

References 1-2 - 90.80% Coverage

My cgm failed 4 days early and I didn't feel the hypo I had walking to the library with School. Luckily my Mum came with me and her spidey senses were working. She tested my blood sugar with a finger prick and treated the low. I felt very hungry and thirsty and could not concentrate on what the class were doing.

<Files\\1\\Photo 6 voice (child)> - § 2 references coded [88.89% Coverage]

References 1-2 - 88.89% Coverage

My blood glucose sensor failed half way through the week. On a good note though peeling off my CGM feels amazing after having it stuck to my body for a few days. The CGM can annoy me and the tape makes me itchy. It feels like luxury when I am free of it.

<Files\\10\\Photo 1(parent and child)> - § 4 references coded [91.73% Coverage]

References 1-2 - 67.95% Coverage

We love our son's Tandem pump however was massively stressed when we got this malfunction [illegible]

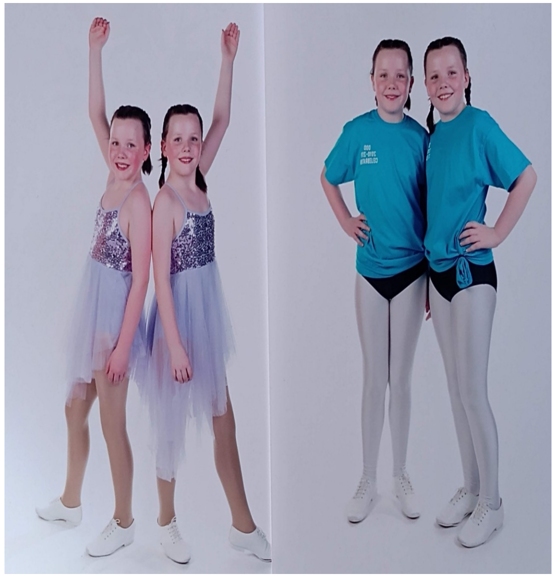

Example of pre coding labels in red and collaborative coding/changes in blue

Submitted narrative data:

Teen: Had dress rehearsal for my Dance show. My mum takes care of my diabetes when I'm dancing so I get to enjoy the show just as much as my twin sister Annalisa. The photographer posed us in such a way my pump doesn't show in the photos as I get very self conscious around new people. I spend most of the dance show without my pump on as I'm moving about too much and have lots of dress changes. I snack on sugar/carb free foods to keep my energy up but avoid high blood sugars as I am on and off stage for 3 hours.

Mum: Dress rehearsal and show is manic. It takes days of planning and I often don't sleep the night before making sure I don't just have all the dance outfits packed but that I have snacks, hypo treatment, all things diabetes and then some more just for good luck. There's usually always something I have forgotten. I often feel like I'm drowning in diabetes supplies but I never let the girls see me stressing. It's happy faces all round so they enjoy showtime. I try keeping * sugars perfect for dance shows so she can enjoy it like everyone else and that usually means her pump is on and off a few times that night. She is usually jumping about all over the place and doesn't notice me attaching and detaching it.

Initial researcher codes/ co-researcher changes:

Parental gatekeeping– diabetes support whilst teen is active

Hiding pump – visibility of technology/identity

Planning ahead to enable physical activity – Parent has all the burden to help teen

Trying to prevent risk – preventing risk and relying on the tech

Parent easing teen burden of the condition – I try to use the tech without it disrupting

Participant 1
(Male adoelscent, Age 9)

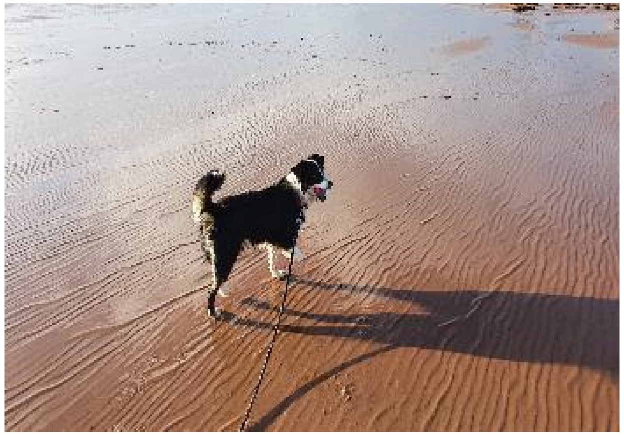

I went for a walk with my Dog when suddenly my pump alarm told me I was going Low. I am so lucky to have a CGM and a new dog.

Analysis codes:
1 Benefits– alarm/alert
Parent agrees
2 Hypoglycaemia whilst walking
Hypo whilst active

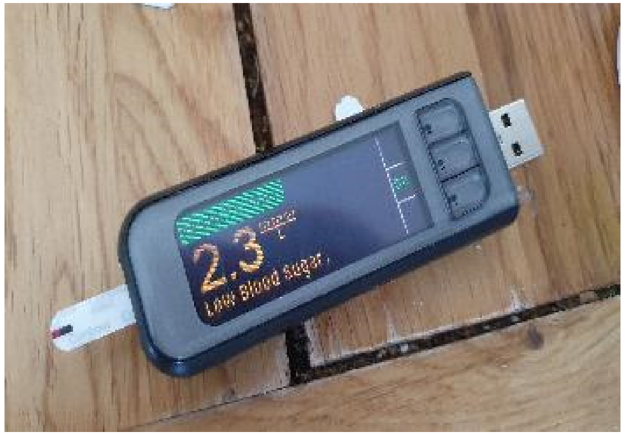

Feeling super hungry after a long walk on the beach. I didn't hear my pump alarm. Turns out I was extremely hypo.

Analysis codes:

1 Technology alerts
2 Alarms not always audible
3 Extreme hunger whilst hypo
Change alerts?
Reliance on alerts

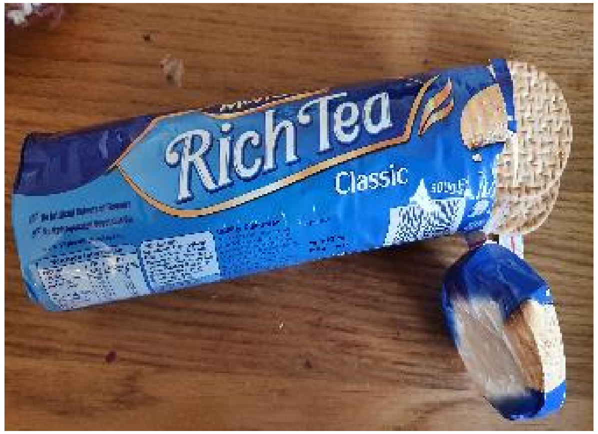

Yesterday I had a "Snackccident" at School after a torturous gym session. I felt extremely tired and hungry. My blood sugars were low but not hypo. I forgot to take insulin. My classroom assistant was not happy I stole the snacks.

Analysis codes:
1 Extreme hunger whilst hypo
2 Forgetfulness
When I’m not there / moving away from me

Nvivo coding – classifying data, naming themes

Quick Access
- Files
- Nodes
- Memos

Data
- Files
  - 1
  - 10
  - 11
  - 12
  - 13
  - 14
  - 15
  - 16
  - 17
  - 18
  - 19
  - 2
  - 20
  - 3
  - 4

Nodes

Name	Files	References
benefits of technology - usability & psychosocial	32	34
communication & immediate support	9	9
ease of psychological burden	4	4
passivity & glanceability	22	23
complexity and difficulty	26	26
transitions from parent	2	2
Uncertainty and PA induced hypos	13	14
Visibility of T1	4	4
Emotional impact	55	60
confidence through support	10	10
parental burden in facilitation	14	14
parental sleep disruption	15	15
resilience & positivity	15	15
teen negative emotions	19	19
reliance and risk	29	31
Tech fails & severe life risk	35	38
trust& vigilance	4	5

Nvivo coding – classifying data, naming themes

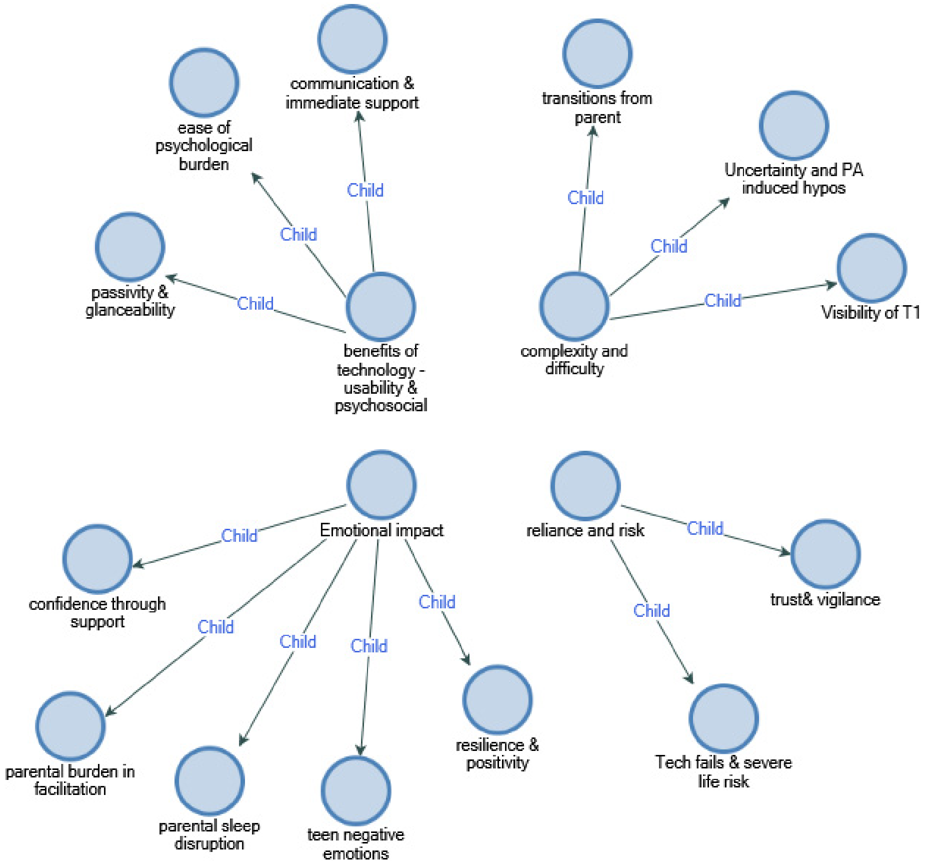

NVivo map showing movement from sub-theme to higher order themes, after 2nd cycle of coding.

Practices involved research team congruence and agreement across all data, sharing all images and narratives.
